# A content analysis of the Orbeez^®^ Gel Blaster injury challenge on TikTok

**DOI:** 10.1186/s40621-024-00557-7

**Published:** 2025-02-18

**Authors:** Hannah P. Schneider, Jamie M. Reedy, Rebecca J. McAdams, David I. Swedler, Jennifer A. Manganello, Kristin J. Roberts, Leah K. Middelberg, Lara B. McKenzie

**Affiliations:** 1 Center for Injury Research and Policy, The Abigail Wexner Research Institute at Nationwide Children’s Hospital, 700 Children’s Drive, Columbus, OH 43205 USA; 2 Department of Health Policy, Management, and Behavior, School of Public Health, University at Albany, 1400 Washington Ave, Albany, NY 12222 USA; 3 ForHealth Consulting at UMass Chan Medical School, 333 South Street, Shrewsbury, MA 01545 USA; 4 Department of Pediatrics, College of Medicine, The Ohio State University, 370 W. 9th Ave, Columbus, OH 43210 USA; 5 Division of Epidemiology, College of Public Health, The Ohio State University, 250 Cunz Hall, 1841 Neil Ave, Columbus, OH 43210 USA; 6 Department of Pediatrics, Division of Emergency Medicine, Nationwide Children’s Hospital, 700 Children’s Drive, Columbus, OH 43205 USA

**Keywords:** Social media, TikTok, Elaboration likelihood model

## Abstract

**Background:**

TikTok is one of the fastest-growing social media platforms. With 50 million active daily users in the United States, videos on TikTok have the opportunity to reach an exceptionally large audience. It is of concern that some of these videos may be harmful, especially if they encourage dangerous or risky behavior that can increase injury risk. This is common for social media challenges, where people are encouraged to participate in and record a task and then post it on social media. The “Orbeez Challenge” was a challenge that went viral in 2022 and encouraged viewers to shoot water beads with Gel Blaster guns at others. The purpose of this study was to describe content for the “Orbeez Challenge” on TikTok, informed by pathways of the Elaboration Likelihood Model (ELM).

**Methods:**

This study conducted a content analysis of TikTok videos related to the Orbeez challenge identified between October 5–11, 2022. A codebook was created by using abductive code generation, driven by an inductive iterative review of a sample of videos not included in the final study sample, and deductive code development to collect elements of the ELM.

**Results:**

There were 125 videos in the final sample. Collectively, these videos had over 255 million views. Most (70.0%) of the creators were laypersons. Text (*n* = 97; 77.6%) and music (*n* = 83; 66.4%) were frequently present in the videos. Individuals were primarily shooting (47.2%) or loading (25.6%) the Gel Blaster. Nearly half (46.3%) of the content creators had between 10,000 and 500,000 followers. Most videos (*n* = 109; 87.2%) did not depict any injury prevention precautions, but of the 15 videos (12.0%) that did show injury prevention precautions, 14 (93.3%) were for eye protection.

**Conclusions:**

This study characterized the content of the TikTok videos associated with the viral “Orbeez Challenge” and showed that TikTok videos could be characterized with guidance from a health communication theory. Despite their increased injury risk, the videos had high engagement and were most frequently posted by laypersons. This research presents an opportunity for countering injury challenges on social media and can guide injury professionals in designing and improving virtual health education campaigns.

**Supplementary Information:**

The online version contains supplementary material available at 10.1186/s40621-024-00557-7.

## Introduction

With approximately 4.7 billion users worldwide, social media is one of the most popular ways to share and exchange ideas, interests, and information [1]. TikTok is one of the fastest-growing platforms, with 834 million users [2]. The United States has the largest TikTok audience (135 million total users, 50 million active daily users), with the highest portions of users aged 10–19 (25.0%) and 20–29 years (22.4%) [2]. The ease and immediacy of content sharing on TikTok, along with the platform’s popularity, has enabled many videos to go “viral,” meaning the video has an extremely high engagement (i.e., number of views, likes, and shares), reaching a large audience.

Unfortunately, the content of videos can be harmful if they encourage dangerous or risky behavior. This is common for social media challenges, where people are encouraged to participate in a task, record it, and then post it to social media. For example, the “Cinnamon Challenge” encouraged viewers to consume a spoonful of cinnamon without drinking any liquids, which often resulted in coughing, choking, and gagging, with extreme cases of ingestion requiring poison control consultations and emergency room visits [3]. Other dangerous challenges included the “Kiki Challenge,” where people jumped from a moving car and danced while the car continued to drive [4], and the “Benadryl Challenge,” where individuals consumed extremely high doses of the drug Benadryl (diphenhydramine) to induce a “high” effect and share content of the resulting behavior [5]. These challenges and others have resulted in severe injuries and fatalities [5, 6]. 

**Fig. 1:**
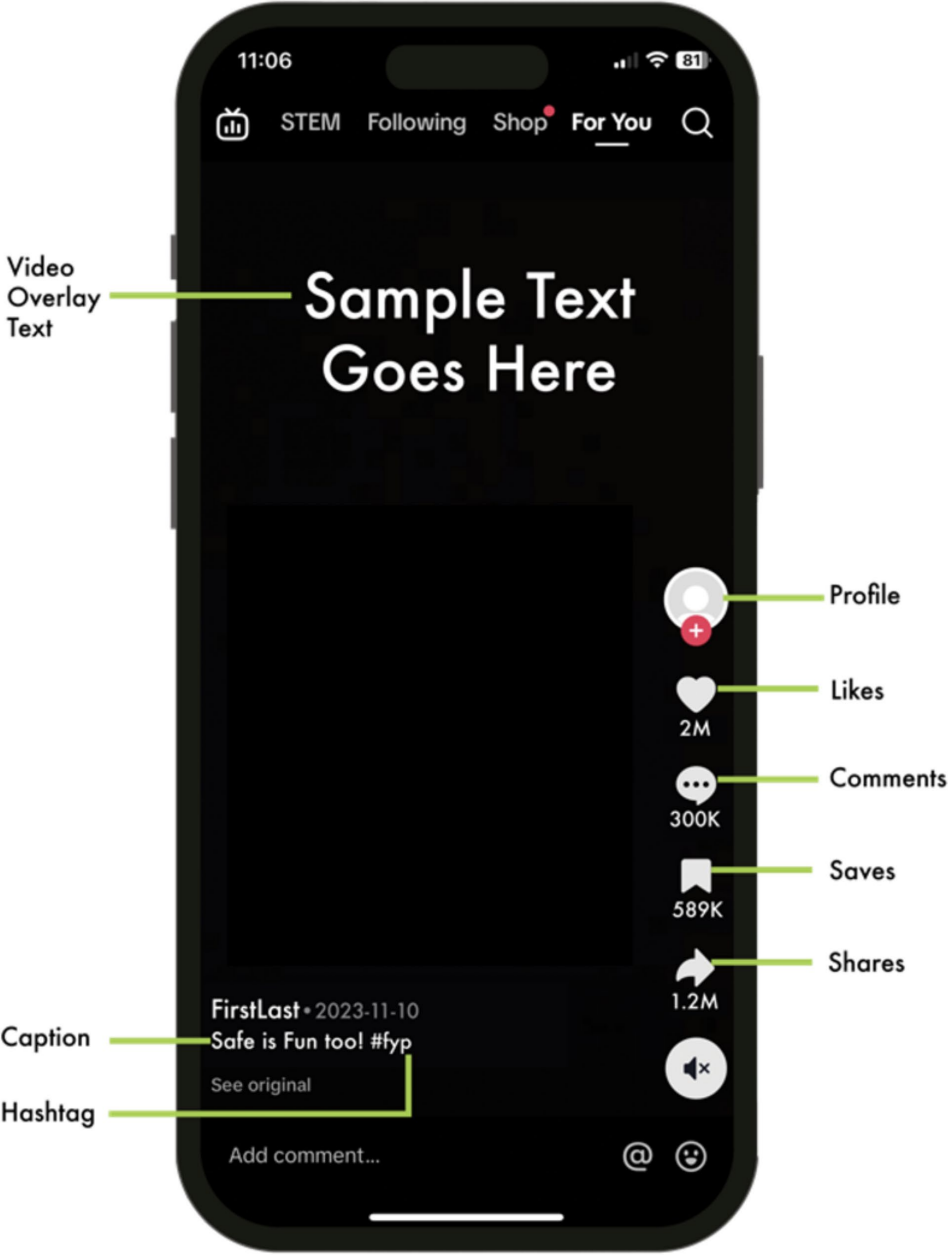
Depiction of a general TikTok video

The present study investigated the “Orbeez Challenge,” also known as the “Gel Blaster Challenge,” which went viral (i.e., the video became popular and spread widely across social media) on TikTok in 2022. Despite the challenge name, the Orbeez brand is not affiliated with the Gel Blaster brand or this challenge [7]. The challenge involves shooting water-based beads (“gellets”) from air- or battery-powered toy guns (Gel Blasters) at others (often random civilians with no safety gear) [7]. It has resulted in many negative consequences, including numerous injuries to shooters and bystanders, as well as arrests and other law enforcement involvement [7]. An exemplifying case presented by our study team involves a male teen who presented to the emergency department (ED) with an eye injury after being shot with a Gel Blaster while participating in the TikTok challenge (Fig. 1). The patient had immediate blindness, and an ophthalmologic exam indicated an almost complete (95%) traumatic hyphema. The patient required surgery to drain blood from the eye’s anterior chamber and fortunately regained normal vision after several months. This patient’s injury is consistent with two studies collectively reporting 36 cases of significant ocular trauma from gel pellet projectiles over the past several years [8, 9]. 

The Elaboration Likelihood Model (ELM) may help explain participation in these challenges and account for factors in viewer decision-making. The ELM is a communication theory that describes how people process content along the cognitive pathway from views to action (i.e., behavior) via two pathways: central route (i.e., the credibility and expertise of the content creator, message quality, and the viewer’s existing attitudes about the content) and peripheral route (i.e., the video’s aesthetics and environment, attractiveness and likeability of the creator, and the viewer’s perceived similarity with the creator) [10–12]. 

Prior literature posits why individuals may participate in these dangerous challenges despite obvious and significant health and safety risks [13–21], but few studies have described how challenge content is presented on social media and what, if any, risk information is portrayed [22, 23]. Given the prevalent TikTok usage among younger age groups and the potential for severe injury during the Orbeez challenge, knowing the posted content for this challenge and applying the ELM to the content will help us to understand the pathway leading to risky behaviors and to strategize ways for minimizing associated harms with this challenge and others. The current study characterized content for the “Orbeez Challenge” on TikTok, informed by the pathways of the ELM. No prior published studies have used the ELM to inform and describe TikTok challenges. A secondary goal was to develop a framework for injury and health professionals to evaluate TikTok challenge content using an established communication theory. Our research questions were: (1) How is the Orbeez Challenge characterized on TikTok?, (2) How can the ELM be applied to the Orbeez Challenge on TikTok?, and (3) How is injury content for the Orbeez Challenge portrayed on TikTok?

## Methods

### Study sample

Following past methodologies [24], a research team member (HPS) identified and downloaded 125 videos between October 5th and 11th, 2022, using a new TikTok account and incognito page to scroll through publicly available posted videos. The inclusion criteria for this study were that the videos must be in English and show or reference the Gel Blaster toy. Virality of video was not an inclusion criterion. The exclusion criteria were videos not in English and did not reference the Gel Blaster. Duplicate videos were also not included; the researcher would skip the duplicate video and move to the next unique video. Due to the common practice of using misspelled hashtags to avoid blocking by TikTok safety features, the present study selected TikTok videos by searching five of the most frequent Orbeez Challenge-related hashtags: #orbezchallenge, #gelblaster, #splatterball, #splatballguns, #splatrball (*n* = 25 unique videos for each hashtag). Videos were downloaded or screen recorded/screenshotted, assigned, and stored with a unique identifier. The saved files included content creator profiles and to-date likes, comments, shares, and views. This research was exempt from IRB review from Nationwide Children’s Hospital because it is not considered human subjects research.

## Codebook development

A codebook was created using abductive code generation, driven by deductive code development to collect elements of the ELM and an inductive, iterative review of a small sample of videos not included in the final study sample. The Qualtrics (Qualtrics 2020, Provo, UT) platform was used for data collection, and RStudio (Posit Software, PBC, Boston, MA) and SAS version 9.4 (SAS Institute, Inc., Cary, NC) were used for the analysis (Appendix 1 for final codebook). Two authors (HPS, JMR) were assigned a random 25% sample of videos (every 5th video of each hashtag) to double code for reliability measurement, reaching an acceptable agreement between coders (Cohen’s Kappa = 88%). One author (HPS) then completed the coding of the remaining videos.

The codebook was separated into seven categories [25–27], each with a varying number of variables: (1) creator, (2) interactions, (3) people, (4) Gel Blasters, (5) characteristics, (6) ELM-central route, and (7) ELM-peripheral route (Table 1). For the creator category, the study team collected information regarding the creator type (i.e., layperson or news source) and if the creator was verified at the time of video collection (yes/no). The four variables in the interaction category collected information on the number of views, likes, shares, and comments for the video at the time of download. The people category had four variables, including if the person in the video is using or holding a Gel Blaster (yes/no), how the Gel Blaster was used by the people in the video (i.e., shooting at other people, shooting at inanimate objects, loading, and other non-shooting activity such as decorating), if you can see the face of the person using the Gel Blaster (yes/no), and the perceived sex of the person holding the Gel Blaster (male, female, or cannot tell). The Gel Blaster category collected information on whether the Gel Blaster was shown or mentioned (yes/no), if any of the Gel Blaster components were for sale (yes/no), and if there is a mention of how to learn more about what is taking place in the video (yes/no). The characteristics section of the codebook collected data on duration of video, presence of music, additional text and/or voiceover (yes/no), presence of video caption (yes/no), and video type (original, where the creator made the video themselves; stitch, where someone else created the video content; duet where the content creator recorded themselves with someone else’s video side-by-side; or other).

**Table 1:** Data elements collected from the *n* = 125 Orbeez Challenge-related TikTok videos

Variable Category	Defintion	Variables
Creator	The individual who posted the video content	Creator type; Verified creator
Interactions	An indication of engagement with the video	Number of views, likes, shares, comments
People	Descriptions of individuals who were in the video, including those who could be seen and those who could not be seen (i.e., holding the camera)	Holding a Gel Blaster; How people were using the Gel Blaster; Face of person using the Gel Blaster is shown; Perceived sex of the person holding the Gel Blaster
Gel Blasters	How the Gel Blaster was portrayed in the video	Gel Blaster shown/mentioned; Gel Blaster components for sale; Mention of how to learn more about what is taking place in the video
Characteristics	Descriptors of the video	Video duration; Presence of music, additional text, and/or voiceover; Presence of video caption; Video type
ELM- Central Route	Factors related to the central route of the ELM	Main theme of the video; Presence of distractions to the main theme; Video tone; Presence of a call to action; Type of injury prevention precaution; Who was utilizing the injury prevention gear
ELM- Peripheral Route	Factors related to the peripheral route of the ELM	Attractiveness of the content creator; Creator within targeted TikTok viewing demographic (18–34 years of age); Presence of fun; Presence of a specific brand of Gel Blaster; Presence of professional production elements; Influencer level

There were six variables in the central route category. The main theme of the video was coded as either product promotion; playing: shooting; playing: loading, demoing, consequence awareness, and selling an item. We collected information on the presence of distractions to the main theme (e.g., yes, where the music does not match the tone of the video, no distractions), the overall tone of the video (positive, negative, neutral, unclear), and the presence of a call to action (yes/no). Injury prevention precautions were coded if a specific type of injury prevention precaution was collected (i.e., eye protection), and who was utilizing the protective gear.

Peripheral route factors included the attractiveness of the content creator, which was defined via a validated attractiveness scale [28] and delineated into groups of attractive, not attractive, and unclear for this study. We also identified if the creator appeared to be within the most popular TikTok viewing demographic age range of 18–34 years (yes, no, unclear). We determined if there were any characteristics of fun in the video (yes, including playing, laughing, and cheering, or no). We recorded if a specific brand of Gel Blaster was in the video (yes/no), and if the video has any professional components (yes, including being filmed with professional equipment, or no). Additionally, the influencer level was separated into eight groups [29] ranging from not an influencer (< 1,000 followers) to mega influencer (1,000,000-< 5,000,000).

### Data analysis

We conducted a quantitative content analysis to code and analyze the Orbeez Challenge TikTok content. Our goal in the analysis was to confirm that we could successfully enter and analyze data on TikTok injury challenges, not to provide statistical evidence for ELM pathway factors in determining individual or group attitudes or behavioral intentions. In demonstrating the ELM as a framework for data collection, we also recognize that not all videos contain content for every data element in the codebook. In some cases, video elements covered by the codebook were lost due to the archiving process. Acknowledging these goals, we conducted a descriptive content analysis without statistical testing of frequencies. We calculated total engagement rate as: [30]

$$\begin{aligned}&\:TikTok\:Engagement\:Rate\\&\quad = \left(\frac{\left(Total\:Likes\right)+\left(Total\:Comments\right)+\left(Total\:Shares\right)}{\left(Total\:Views\right)}\right) * 100 \end{aligned}$$

## Results

As of download date, the 125 videos in the sample had been viewed over 255 million times, liked over 25 million times, shared over 400,000 times to others (Table 2), and had an engagement rate of 10.2%. The videos had an average length of 33.2 s (SD = 39.2). The shortest video was 6 s, while the longest was just over 5 min (301 s). Nearly 94% (*n* = 117; 93.6%) of the videos were original, meaning the creators made the videos themselves. Over 70% of creators (*n* = 88; 70.4%) were laypersons, 34 (27.2%) were Gel Blaster representatives, two (1.6%) were news sources, and a single (0.8%) creator self-identified as a lawyer. Almost all (*n* = 123; 98.4%) of the creators were not TikTok verified.

**Table 2:** Descriptive data for views, likes, shares and comments for the 125 TikTok videos identified with Orbeez challenge-related hashtags

	Total	Minimum	Median	Maximum
Views	255,512,098	6,818	392,700	51,400,000
Likes	25,404,025	77	28,200	6,900,000
Shares	402,730	3	370	77,400
Comments	198,591	5	224	48,400

Text (*n* = 97; 77.6%), music (*n* = 83; 66.4%), and voiceovers (*n* = 47; 37.6%) were frequently present in the videos. Most videos (*n* = 96; 76.8%) utilized captions on the videos in addition to hashtags located at the bottom of the post in a text section, as illustrated in Fig. 1.

At least one Gel Blaster was shown or referenced in all but two videos (98.4%). In the 123 videos where Gel Blasters were seen or mentioned, they were used to shoot at inanimate objects (*n* = 42; 34%), loaded (*n* = 36; 29%), shot at people (*n* = 27; 22%), or some other non-shooting activity (*n* = 27; 22%). A person was using or holding a Gel Blaster in 88.8% (*n* = 111) of the videos. Of those, the majority of users’ faces are obscured (*n* = 62, 55.9%). However, in the 49 (44.1%) videos where the user’s face is identifiable, most were male (*n* = 40, 81.6%). Two videos (2%) had links to purchase Gel Blaster components, and six videos (5%) mentioned how to learn more about what was being depicted in the video.

## Elaboration likelihood model results

### Central route

Thirteen main themes were seen in the videos, as described in Supplemental Table 1. The most common main themes were an individual playing with the Gel Blaster by shooting it (*n* = 59; 47.2%) and an individual playing with the Gel Blaster by loading it (*n* = 32; 25.6%). Only 18 videos mentioned any consequences of using a blaster, with consequences falling into non-exclusive categories of law enforcement (*n* = 14), injury (*n* = 5), and school (*n* = 2). Most videos (*n* = 109; 87.2%) did not depict any injury prevention precautions, but of the 15 videos (12.0%) that showed injury prevention precautions, 14 (93.3%) were for eye protection. The engagement rate of videos with and without injury prevention content was 9.2% and 10.4%, respectively. Over three-quarters of videos (*n* = 103; 82%) did not have any distractions from the main theme; however, the remaining videos had distractions that included visuals that did not match the audio (*n* = 12; 9.6%) or music that did not match the tone of video (*n* = 6; 4.8%). The overall tone of the videos was mostly positive (i.e., fun) (*n* = 69; 55.2%). Of the 26 videos (20.8%) that included a specific “call to action” most (*n* = 15; 57.7%) were requests for viewers to follow the content creator.

## Peripheral route

When the content creator could be identified, 47.1% (*n* = 24) were attractive, 27.5% (*n* = 14) not attractive, and 25.5% (*n* = 13) attractiveness unclear. We found that over 80% (*n* = 41) of identifiable creators appeared to be in the target demographic age range (18–34 years). A total of 40.0% (*n* = 50) of the videos had some aspect of fun, including playing with the Gel Blaster (*n* = 38), laughing (*n* = 22), and decorating (*n* = 7) the Gel Blaster. Most videos (*n* = 77; 61.6%) did not highlight a specific brand of Gel Blaster, and nearly all (*n* = 11; 97.6%) videos did not have a professional component. Among the 108 videos with a known number of followers, two creators (1.9%) had more than 1 million followers, 10 (9.3%) had between 500,000 and 1,000,000 followers, 50 (46.3%) had between 10,000 and 500,000 followers, 34 (31.5%) had between 1,000 and 10,000 followers, and 12 (11.1%) had fewer than 1,000 followers (Table 3).

**Table 3:** Number and percent of TikTok video creators based on their influencer level

Influencer Level	Number of Followers	*n*	%
Mega influencer	$\:\ge\:$1,000,000	2	1.9
Macro influencer	500,000 - <1,000,000	10	9.3
Mid-tier influencer	50,000 - <500,000	20	18.5
Micro-influencer	10,000 - <50,000	30	27.8
Nano-influencer	1,000 - <10,000	34	31.5
Not an influencer	< 1000	12	11.1

## Discussion

The purpose of this study was to describe TikTok videos associated with an injury risk challenge and apply the ELM to explain why individuals may be inclined to participate in this challenge. The study findings illustrated remarkably high video engagement with the Orbeez Challenge but minimal injury prevention consequences, precautions, or messaging despite known injuries associated with the challenge. When applying the ELM, the videos showed that most creators were laypeople and minor influencers within the targeted TikTok demographic playing with a Gel Blaster. Given the limited number of injury prevention and consequence videos, the popularity of the videos is guided by aspects of the peripheral route.

Our study is novel; to our knowledge, this is the first of its kind to apply the ELM to TikTok videos. Furthermore, past research indicates that social modeling and learning may explain the reasoning for why social media users participate in dangerous challenges [13, 16, 31, 32], but there is a lack of information on what actual content characteristics are attracting users on TikTok to watch the challenges. Our study is also novel because it investigates these characteristics.

Aligning with prior studies that have examined viral challenges on other social media platforms [31–33], we found that there is minimal injury prevention content and safety messaging associated with the Orbeez Challenge, underscore other novel factors of our study. Our findings are consistent with previous research that peripheral route factors, such as source attractiveness and source relatedness, are often enticing for users on social media platforms [34]. This study may help researchers design health education interventions on TikTok to mitigate the dangers of challenges by emphasizing peripheral route factors while also incorporating central route factors like argument quality. Researchers can use knowledge of the ELM to appropriately create attractive safety messaging content on the platform that will reach and connect with the target audience. Further, future studies can examine the effectiveness of using the ELM factors to counteract these risky social media challenges.

Despite most videos coming from unverified creators and lower-tier influencers (98%), video engagement was exceptionally high. These videos amassed over 255 million views at the time of video download and had a 10.2% engagement rate, almost twice the average for TikTok in 2022 (5.7%) [35]. This supports the trend on TikTok where the “next-door neighbor” can be viral the next day and that behavioral influence, including participation in challenges, does not need to come from celebrity influencers or verified creators [36]. 

The Orbeez Challenge is considered to be associated with injuries [8, 9], yet our results demonstrate that most videos lacked presentation of any safety precautions, risk messaging, or prosocial suggestions. Only 15 videos had any injury-prevention content, and only five showed evidence of injury consequences. Of the videos demonstrating injury precautions, the majority only included eye safety goggles with no other precautions or recommendations. Given that the case study had a significant eye injury that may have been prevented with proper eye protection, it is concerning that so few videos demonstrated eye safety and failed to call viewers to action on eye protection or other safety precautions.

The engagement rate of both videos with and without injury prevention content was similar (9.2% vs. 10.4%, respectively), which is higher than the reported average [35]. These above-average engagement rates demonstrate the popularity of Orbeez challenge videos, regardless of injury prevention content. Our sample found no videos from trusted health officials, medical providers, or injury prevention specialists. Given the absence of trusted messaging and poor engagement of videos from non-peers, behavioral motivation for this challenge seems to be driven by the peripheral factors often considered unimportant in health message crafting.

This study highlights that the ELM can provide insight into why injury challenge videos proliferate despite evidence of risk. Previous literature indicates that aspects of the peripheral route may have more influence on TikTok-associated behaviors designed to increase engagement than those of the central route [34]. Personal relevance, entertainment value, and attention-seeking are key motivators for viral behaviors. Furthermore, the emotional experience and perceived closeness to the source help to create social connections that increase views and actions [37]. This experience-sharing effect is often driven by social identification and source attractiveness [28, 38]. Our findings align with this, as many creators in our sample were identified as part of the typical TikTok age demographic (adolescent – mid-thirties), with almost half subjectively identified as attractive. Additionally, many videos had some element of fun, likely contributing to the above-average engagement.

The ELM was also valuable in understanding viewers’ motivation to engage in risky behaviors. Past research indicates that viewers participate in challenges without understanding the risks associated with the behavior, but instead, they do so based on factors like engaging in fun activities or mimicking attractive sources to participate in a social experience [13, 15]. Furthermore, a call to action is not needed to encourage people to engage in risky behavior they see on social media; instead, behavioral motivation is driven by other factors, such as peripheral route attributes of experience-sharing, media richness, and having fun. This is supported in our study, where although nearly 80% of the videos lacked any call to action to participate in the challenge, and very few video themes revolved around consequences or safety, engagement was high.

Given our findings, several potential practice implications and strategies exist. Parents can be educated about TikTok challenges that are potentially dangerous in order to engage in discussions with their children about potential consequences, agreeing on safe use and avoiding risky behaviors. TikTok should focus on continued research and implementation of safety regulations, including blocking dangerous content or material that violates community standards, attaching content warnings to risky activities, sharing links to medical or self-help hotlines, and encouraging informed decision-making. Injury prevention specialists should also consider partnerships with individuals in the TikTok age demographic who are considered credible on the platform to further promote engagement. Stories from peers, like the individual in our case study, also present a unique opportunity for sharing lived experiences with viral challenges and the associated consequences. Health professionals have the opportunity to develop educational modules for parents and adolescents focused on media literacy, social media trends, and risk prevention. Safety messaging campaigns can be developed to mitigate participation in potentially risky challenges. They should apply the peripheral factors important for video engagement, such as attractiveness and fun [39]. 

### Limitations and strengths

This study had some limitations. Due to the novel application of the ELM to TikTok, the researchers operationalized concepts for the central and peripheral routes to a new medium with no validated cases. The video content varied broadly because of the nature of user-generated videos and creator-applied hashtags. Furthermore, one of the key peripheral route factors, attractiveness, was challenging to define for coders due to its subjectiveness. The team attempted to overcome this obstacle by operationalizing attractiveness with strategies used in prior literature [28]. Additionally, the age range of the content creator is a limitation due to its subjectivity. The study team elected to maintain this ELM factor based on the established importance it has historically played on attitude and behavior change and felt coding reliability was adequate based on the high interrater agreement. Another limitation of this study is that it only focused on one viral challenge on one social media platform. Future research is needed to determine if the ELM can be applied to additional social media challenges and on other platforms.

Additionally, some organizations banned TikTok before the time of video downloads. Only a fraction of our sample videos included risk messaging, safety precautions, and injury consequences, and there was no representation by any healthcare professional or injury prevention creators. The TikTok ban could have influenced the absence of reliable health information from trusted sources. Despite these limitations, this study has many strengths. Importantly, this study provided a framework for applying the ELM to risk and safety content videos on TikTok. The researchers applied rigorous and sound strategies to create the codebook. Also, the interrater agreement score was high, indicating high coding precision. Finally, the authors used a Google Trends analysis of the Orbeez Challenge search term and found that the challenge was most searched for in 2022 [31], corresponding to the time frame for video collection for our study.

## Conclusion

This study characterized the content of TikTok videos related to the Orbeez Challenge by using a health communication theory. Our study found that despite the known association with increased risk of injury, few of these videos had risk messaging or injury prevention content. The videos had high engagement, which demonstrated that behavior may be driven more strongly by peripheral route factors than central route factors. Overall, this research presents an opportunity for countering injury challenges on social media and can guide injury professionals in designing and improving virtual health education campaigns.

## Electronic supplementary material

Below is the link to the electronic supplementary material.

Supplementary Material 1

Supplementary Material 2

## Data Availability

The datasets used and analyzed during the current study are available from the corresponding author on reasonable request.

## Abbreviations

ELM: Elaboration Likelihood Model
ED: Emergency Department
