# Supplementary material for: A content analysis of the Orbeez® Gel Blaster injury challenge on TikTok
Source: Inj Epidemiol. 2025 Feb 18;12:9. doi: 10.1186/s40621-024-00557-7 (PMC11834493; doi:10.1186/s40621-024-00557-7)
Supplement: Supplementary file 1 — Supplementary Material 1 [file 40621_2024_557_MOESM1_ESM.docx]

**Supplemental Table 1.** Main themes of the n=125 Orbeez Challenge-related TikTok videos

| **Main theme** | **n** | **%** |
| --- | --- | --- |
| Playing: shooting | 59 | 47.2 |
| Playing: loading | 32 | 25.6 |
| Decorate/enhance the gel blaster | 20 | 16.0 |
| Product promotion | 15 | 12.0 |
| Consequence awareness: Law enforcement | 14 | 11.2 |
| Consequence awareness: injury observance | 5 | 4.0 |
| Demo: instructions | 4 | 3.2 |
| Testing a theory | 3 | 2.4 |
| No theme | 3 | 2.4 |
| Consequence awareness: school officials | 2 | 1.6 |
| Consequence awareness: individual retaliation | 1 | 0.8 |
| Selling an item | 1 | 0.8 |
| Other (including holding the gel blaster) | 28 | 22.4 |
