# Supplementary material for: A content analysis of the Orbeez® Gel Blaster injury challenge on TikTok
Source: Inj Epidemiol. 2025 Feb 18;12:9. doi: 10.1186/s40621-024-00557-7 (PMC11834493; doi:10.1186/s40621-024-00557-7)
Supplement: Supplementary file 2 — Supplementary Material 2 [file 40621_2024_557_MOESM2_ESM.docx]

**APPENDIX 1 CODEBOOK**

| **Q** | **VARIABLE NAME** | **QUESTION TEXT** | **RESPONSE OPTIONS** | **NOTES** |
| --- | --- | --- | --- | --- |
| Q1 |  | Data entry personnel initials | Free text |  |
| Q2 |  | Data entry date | Free text |  |
| Q3 |  | Video ID | Free text |  |
|  | Section 2 (Creator) |  |  |  |
| Q4 | CREATE1 | Who is the creator? | 1. Health profession (physician, nurse, injury professional) 2. Gel blaster rep (someone trying to sell the gel blaster or products) 3. News source (any news posting/warning about gel blaster) 4. Layperson (someone who is just a person using the gel blaster) 5. Law enforcement (police) 6. Can’t tell 7. Other (free text) |  |
| Q5 | CREATE2 | Is the creator verified? | 1. No (0) 2. Yes (1) |  |
|  | Section 3 (Interactions) |  |  |  |
| Q6 | INTERACT1 | How many views does the video have? | Free text |  |
| Q7 | INTERACT2 | How many likes does the video have? | Free text |  |
| Q8 | INTERACT3 | How many forwards does the video have? | Free text |  |
| Q9 | INTERACT4 | How many comments does the video have? | Free text |  |
| Q10 | INTERACT5 | What date was the video posted? | Free text |  |
|  | Section 4 (Gel Blaster) |  |  |  |
| Q11 | BLASTER1 | Is the gel blaster shown/mentioned? | 1. No (0) 2. Yes (1) |  |
| Q12 | BLASTER2 | What is the topic of the TikTok? (Check all that apply) | 1. News warning 2. Product promotion 3. Playing: shooting 4. Playing: loading 5. Demo: instructions on how to use, shoot, or load (explicitly stating directions) 6. Safety precautions 7. Law enforcement involvement 8. None 9. Other (free text) |  |
| Q13 | BLASTER3 | Is the gel blaster, ammo, or components for sale via link (or referenced as “link in bio”)? | 1. No (0) [SKIP TO BLASTER5) 2. Yes (1) [GO TO BLASTER4) |  |
| Q14 | *BLASTER4* | *If there is a link, what is for sale? (Check all that apply)* | 1. *Gel blaster* 2. *Ammo (Orbeez, “gellets”)* 3. *Safety glasses* 4. *Charger* 5. *Other (free text)* |  |
| Q15 | BLASTER5 | Is there mention of information to learn more about what is taking place in the video? (another user profile, Instagram profile, link, etc.) | 1. No (0) 2. Yes (1) |  |
|  | Section 5 (Characteristics) |  |  |  |
| Q16 | CHARAC1 | How long is the duration of the video? (minutes:seconds) | Free text |  |
| Q17 | CHARAC2 | Are the following present? (Check all that apply) | 1. Music [SKIP TO CHARAC3] 2. Additional text (any words written on the actual video; may include closed captioning) [GO TO CHARAC2A] 3. Voiceover [SKIP TO CHARAC3] 4. None |  |
| Q18 | *CHARAC2a* | *What is the additional text? (Check all that apply)* | 1. *Closed captioning (subtitles; text that is voiced) [SKIP TO CHARAC3]* 2. *Words written that overlay the video (not spoken; for example: “SAFE TOY”) [GO TO CHARAC2B]* |  |
| Q19 | *CHARAC2b* | *Please write the words overlaying the video:* | *Free text* |  |
| Q20 | CHARAC3 | What type of video is it? | 1. Original (creator made the video themselves) 2. Stitch (someone else’s content on your video, can clip shorter sections, green-screen another user’s video) 3. Duet (recording yourself with someone else’s video side by side) 4. Other (free text) |  |
| Q21 | CHARAC4 | Is there a caption on the video besides the hashtags? (caption text located on the bottom of the post; words without the “#”) | 1. No (0) [SKIP TO PEOPLE1] 2. Yes (1) [GO TO CHARAC5] 3. Not able to view AT ALL on screenshot [SKIP TO PEOPLE1] |  |
| Q22 | *CHARAC5* | *If there is a caption, what is it? *If full caption is not visible in the screenshot used for abstract, please write as much as can be seen. Then write “END”** | *Free text* |  |
|  | Section 6 (People) |  |  |  |
| Q23 | PEOPLE1 | Is there a person in the video using/holding a gel blaster? (gel blaster is actually seen on screen) | 1. No (0) [SKIP TO CENTRAL1] 2. Yes (1) [GO TO PEOPLE2) |  |
| Q24 | PEOPLE2 | How is the gel blaster being used? (Check all that apply) | 1. Shoot at other people [GO TO PEOPLE2A] 2. Shoot at inanimate objects [SKIP TO PEOPLE3] 3. Shoot at animals [SKIP TO PEOPLE3] 4. Loading [SKIP TO PEOPLE3] 5. Non-shooting activity (decorating, enhancing, unboxing) [SKIP TO PEOPLE3] 6. Other (free text) [SKIP TO PEOPLE3] |  |
| Q25 | *PEOPLE2A* | *How many people are being shot at?* | 1. *1* 2. *2* 3. *3* 4. *More than 3* 5. *Unclear* |  |
| Q26 | *PEOPLE2B* | *Does the person(s) being shot appear to know the shooter(s)?* | 1. *No (0)* 2. *Yes (1)* 3. *Unclear (2)* 4. *Other (free text)* |  |
| Q27 | *PEOPLE2C* | *Is the person(s) voluntarily being shot?* | 1. *No (0)* 2. *Yes (1)* 3. *Unclear (2)* 4. *Other (free text)* |  |
| Q28 | PEOPLE2D | Can you see the face of the person(s) being shot at? | 1. No (0) [SKIP TO PEOPLE3] 2. Yes (1) [GO TO PEOPLE2E] 3. Unclear [SKIP TO PEOPLE3] 4. Other (free text) |  |
| Q29 | *PEOPLE2E* | *If you can see a face of who is being shot, account for who is shown: (Check all that apply)* | 1. *Mixed group of genders* 2. *All Female* 3. *All Male* 4. *Can’t tell* |  |
| Q30 | *PEOPLE2F* | *If you can see a face of who is being shot, account for who is shown: (Check all that apply)* | 1. *Mixed group of ages* 2. *Child(ren) (1 or more)* 3. *All teens or adults (no children)* 4. *Can’t tell* |  |
| Q31 | PEOPLE3 | Can you see the face of the person using/demoing the gel blaster? | 1. No (0) [SKIP TO CENTRAL1] 2. Yes (1) [GO TO PEOPLE4] |  |
| Q32 | *PEOPLE4* | *If you can see a face of who is using the gel blaster, account for who is shown: (Check all that apply)* | 1. *Female* 2. *Male* 3. *Can’t tell* |  |
| Q33 | *PEOPLE5* | *If you can see a face of who is using the gel blaster, account for who is shown: (Check all that apply)* | 1. *Child* 2. *Teen or adult* 3. *Can’t tell* |  |
|  | Section 7 (ELM Central Route) |  |  |  |
| Q34 | CENTRAL1 | What is the theme of the TikTok? (Check all that apply) | 1. Product promotion (unboxing, reviewing, brand name placement; reasons to purchase without a link) 2. Sell an item (call to buy a specific brand or product; reasons to purchase with a link) 3. Decorate/enhance the gel blaster (modifying the blaster in some way for personal preference, like painting, embellishing, or installing a larger pellet reservoir) 4. Test a theory/rumor or Debunk myths (“It doesn’t hurt to get shot with a gellet”, “how much does it hurt?,” “can I get shot with a whole gel blaster clip and not move?”, “can I get in trouble for using my gel blaster?”) 5. Playing: Shooting 6. Playing: Loading 7. Participate in the challenge (specifically asks the viewer to participate in the challenge next) 8. Demo: instructions on how to use or load (including how to activate gel pellets – must include specific instructions either spoken or displayed via text; sole purpose is educational) 9. Safety precautions/injury prevention (“You should wear goggles when using a blaster,” “You shouldn’t use the blaster in close range to people,” etc.) 10. Consequence Awareness: Injury Observance (showing or discussing an injury resulting from a gel blaster) 11. Consequence Awareness: Law enforcement involvement (LE called to or involved in an active challenge or involved as a result of a call or complaint after the incident) 12. Consequence Awareness: School officials' involvement (a teacher or school official is included or disciplinary action at school is taken) 13. Consequence Awareness: Individual Retaliation (a person who was targeted by a gel blaster issues a warning to the responsible party or retaliates in some way) 14. None 15. Other (free text) |  |
| Q35 | CENTRAL2 | How was the main theme determined? (Check all that apply) | 1. Video 2. Additional text (words written on the video; may include closed captioning) 3. Voiceover 4. Caption (text written on the bottom of the post; not on video) 5. None |  |
| Q36 | CENTRAL3 | What are the distractions to the main theme(s)? (Check all that apply) | 1. Presence of conflicting themes (more than one theme that is in conflict or contrast with each other) 2. Visuals don’t match the audio (for example, the voiceover mentions having fun, but the video depicts someone wincing in pain or upset) 3. Music doesn’t match the tone of the video (music is upbeat and happy while the video shows a negative or precautionary message) 4. Captions/voiceover/speaker words don’t match up (the captions are incorrect or poorly timed, or voiceover or captions don’t match what the speaker appears to be saying) 5. None 6. Other (free text) |  |
| Q37 | CENTRAL4 | Are people shooting the gel blaster in the video? | 1. No (0) [SKIP TO CENTRAL5] 2. Yes (1) [GO TO CENTRAL 4A] 3. Unclear (2) [SKIP TO CENTRAL5] |  |
| Q38 | *CENTRAL4A* | *How many people are shooting a gel blaster in the video?* | 1. *1* 2. *2* 3. *3* 4. *More than 3* 5. *Unclear* |  |
| Q39 | CENTRAL5 | Are there any injury prevention precautions depicted? | 1. No (0) [SKIP TO CENTRAL6] 2. Yes (1) [GO TO CENTRAL5A] 3. Unclear (2) [SKIP TO CENTRAL6] |  |
| Q40 | *CENTRAL5A* | *What injury prevention precautions are shown/mentioned in the video? (Check all that apply)* | 1. *Eye Protection (goggles, facemask, etc. - Does NOT include regular glasses or sunglasses)* 2. *Body/skin protection (this indicates some kind of padding or protective shield, not just long sleeves or long pants)* 3. *Inanimate targets* 4. *Avoid bystanders* 5. *Other (free text)* 6. *None* |  |
| Q41 | *CENTRAL5B* | *If there are injury prevention precautions depicted, who are they for? (Check all that apply)* | 1. *Shooters [Go to CENTRAL5C]* 2. *Other people [Go to CENTRAL5D]* 3. *Shown on video, but NOT worn or used by any person(s) (i.e., a facemask or goggles are shown on the ground while a shooter aims at a target) [SKIP to CENTRAL6]* 4. *Unclear [SKIP TO CENTRAL6]* |  |
| Q42 | *CENTRAL5C-1* | *Please describe the eye protection precautions associated with the shooter in the video.* SELECT OPTION FOR “ALL SHOOTERS” IF THERE IS ONLY ONE SHOOTER IN VIDEO. | 1. *One shooter is wearing goggles or protective eyewear (this does not include regular glasses or sunglasses)* 2. *Some (but not all) shooters are wearing goggles or protective eyewear (this does not include regular glasses or sunglasses)* 3. *All shooters are wearing goggles or protective eyewear (this does not include regular glasses or sunglasses). SELECT IF ONLY ONE SHOOTER IN VIDEO.* 4. *Goggles, facemask, or other protective eyewear shown in video, but not worn by a shooter.* 5. *Unclear* 6. *None* |  |
| Q43 | *CENTRAL5C-2* | *Please describe the protective body gear associated with the shooter in the video.* SELECT OPTION FOR “ALL SHOOTERS” IF THERE IS ONLY ONE SHOOTER IN VIDEO. | 1. *One shooter is wearing protective body gear (this indicates some kind of padding or protective shield, not just long sleeves or long pants)* 2. *Some (but not all) shooters are wearing protective body gear (this indicates some kind of padding or protective shield, not just long sleeves or long pants)* 3. *All shooters are wearing protective body gear (this indicates some kind of padding or protective shield, not just long sleeves or long pants). SELECT IF ONLY ONE SHOOTER IN VIDEO.* 4. *Protective body gear (this indicates some kind of padding or protective shield, not just long sleeves or long pants) is shown in the video, but not worn by a shooter.* 5. *Unclear* 6. *None* |  |
| Q44 | *CENTRAL5C-3* | *Please describe the inanimate target precautions associated with the shooter in the video.* SELECT OPTION FOR “ALL SHOOTERS” IF THERE IS ONLY ONE SHOOTER IN VIDEO. | 1. *One shooter is not aiming the gel blaster at people or animals in video* 2. *Some (but not all) shooters do not aim the gel blaster at people or animals in video* 3. *All shooters do not aim the gel blaster at people or animals in video. SELECT IF ONLY ONE SHOOTER IN VIDEO.* 4. *None* |  |
| Q45 | *CENTRAL5C-4* | *Please describe the bystander precautions associated with the shooter in the video.* SELECT OPTION FOR “ALL SHOOTERS” IF THERE IS ONLY ONE SHOOTER IN VIDEO. | 1. *One shooter does not shoot the gel blaster in close range to other people shown in video* 2. *Some (but not all) shooters do not shoot the gel blaster in close range to other people shown in video* 3. *All shooters do not shoot the gel blaster in close range to other people shown in video. SELECT IF ONLY ONE SHOOTER IN VIDEO.* 4. *None* |  |
| Q46 | *CENTRAL5D-1* | *Please describe the eye protection precautions associated with the other people shown in the video.* SELECT OPTION FOR “ALL PEOPLE” IF THERE IS ONLY ONE OTHER PERSON IN VIDEO. | 1. *One person (not the shooter) is wearing goggles or protective eyewear (this does not include regular glasses or sunglasses)* 2. *Some (but not all) people (not including the shooter) are wearing goggles or protective eyewear (this does not include regular glasses or sunglasses)* 3. *All people (not including the shooter) are wearing goggles or protective eyewear (this does not include regular glasses or sunglasses). SELECT IF ONLY ONE OTHER PERSON IN VIDEO.* 4. *Goggles, facemask, or other protective eyewear shown in video, but not worn by anyone (not including the shooter).* 5. *Unclear* 6. *None* |  |
| Q47 | *CENTRAL5D-2* | *Please describe the protective body gear associated with other people shown in the video.* SELECT OPTION FOR “ALL PEOPLE” IF THERE IS ONLY ONE OTHER PERSON IN VIDEO. | 1. *One person (not the shooter) is wearing protective body gear (this indicates some kind of padding or protective shield, not just long sleeves or long pants)* 2. *Some (but not all) people (not including the shooter) are wearing protective body gear (this indicates some kind of padding or protective shield, not just long sleeves or long pants)* 3. *All people (not including the shooter) are wearing protective body gear (this indicates some kind of padding or protective shield, not just long sleeves or long pants). SELECT IF ONLY ONE OTHER PERSON IN VIDEO.* 4. *Protective body gear (this indicates some kind of padding or protective shield, not just long sleeves or long pants) is shown in the video, but not worn by anyone (not including the shooter).* 5. *Unclear* 6. *None* |  |
| Q48 | CENTRAL6 | Does the content creator intend for the overall tone to be positive negative or neutral? | 1. Positive (upbeat and happy visuals and audio. For example, laughing in the video or exciting music playing) 2. Negative (gloomy or aggressive music/audio or visuals, direct warnings not to do it. For example, police warn against the consequences) 3. Neutral (no distinct tone or vibe, no music, no specific emotions displayed. For example, 1 person unboxing or demoing the blaster without any associated music) 4. Unclear |  |
| Q49 | CENTRAL7 | Is there a call to action? (A call to action is specific instructions to perform some kind of action. Examples may include buying a gel blaster, clicking a link, subscribing to a service, visiting other social media profiles, watching more content, participating in the challenge, or performing safety precautions) | 1. No (0) [SKIP TO CENTRAL8] 2. Yes (1) [GO TO CENTRAL7A] 3. Unclear (2) [SKIP TO CENTRAL8] |  |
| Q50 | *CENTRAL7A* | *What kind of call to action is included? (Check all that apply)* | 1. *A social media call to action (the content creator asks you to click a link, subscribe to a service, subscribe to their Tik Tok profile, watch more of their content, visit their other social media profiles, visit another user's profile, or to participate in the challenge and post to social media) [SKIP TO CENTRAL7C]* 2. *A purchase or retail call to action (asks for you to purchase something shown or mentioned in the video or included in the captions) [GO TO CENTRAL7B]* 3. *An informational or educational call to action (asks for you to click a link to learn more) [SKIP TO CENTRAL7C]* 4. *An injury prevention call to action (asks you to use safety or injury prevention precautions when personally using gel blasters or when gel blasters are used around you) [Skip to 7C]* 5. *A policy or legal call to action (asks for a change in the policies or laws in either direction relating to gel blaster sale or use. Includes asking for LE to “crack down” on toy gun use OR for LE to stop intervening when it is a toy) [Skip to 7C]* 6. *Other (free text) [SKIP TO CENTRAL7C]* |  |
| Q51 | *CENTRAL7B* | *What is for sale?* | *See BLASTER4* |  |
| Q52 | *CENTRAL7C* | *Would the viewer require prior knowledge of some kind that is not depicted or explained in the Tik Tok to act on the video’s call to action? (For example, knowledge of the laws related to toy gun use to avoid law enforcement involvement)* | 1. *No (0)* 2. *Yes (1)* 3. *Unclear (2)* |  |
| Q53 | *CENTRAL7D* | *Does the video include evidence supporting the call to action? (For example, if there is an injury prevention call to action, does the video share visuals of the gel blaster causing an injury)* | 1. *No (0)* 2. *Yes (1)* 3. *Unclear (2)* |  |
| Q54 | CENTRAL8 | Who is the content creator? | See CREATE1 |  |
|  | Section 8 (Peripheral Route) |  |  |  |
| Q55 | PERIPH1 | Is the content creator attractive? (Based on the literature, attractive means: pretty/handsome, has a sophisticated or glamorous image, wears fashionable or “high end” clothing, the viewer would want to resemble the Tik Toker) | 1. No (0) 2. Yes (1) 3. Unclear (2) 4. Creator is not seen in the video (3) [SKIP TO PERIPH3] |  |
| Q56 | PERIPH2 | Does the content creator appear to be part of the most popular TikTok viewing age demographic? (Ages 18-34) | 1. No (0) 2. Yes (1) 3. Unclear (2) |  |
| Q57 | PERIPH3 | Is the content creator verified? | See CREATE2 |  |
| Q58 | PERIPH4 | What level of Tik Tok influencer is the content creator? | 1. Not an influencer: < 1000 followers 2. Nano-influencers: 1,000 - 10,000 followers 3. Micro-influencers: 10,000 - 50,000 followers 4. Mid-tier influencers: 50,000 - 500,000 followers 5. Macro influencers: 500,000 - 1 million followers 6. Mega influencers: 1 million - 5 million followers 7. Celebrity influencers: Over 5 million followers 8. Unknown (potential profile/content creator no longer exists on TikTok) |  |
| Q59 | PERIPH5 | Does the video include a specific brand? (You can see the brand name on the blaster, the brand is said out loud, the brand name is placed on the video using additional text, or the brand name is present in the post caption; DO NOT include the creator's username) | 1. No (0) 2. Yes (1) 3. Unclear (2) |  |
| Q60 | PERIPH6 | Does the video appear to have professional components? (For example, the video was filmed using a tripod/stand or other professional quality camera skills, the video was well-edited, the audio included has a professional sound quality and is cut to match the video) | 1. No (0) 2. Yes (1) 3. Unclear (2) |  |
| Q61 | PERIPH7 | Are the following present in the TikTok? (Check all that apply) | 1. Music [SKIP TO PERIPH7B] 2. Additional text (any words written on the actual video; may include closed captioning) [GO TO PERIPH8] 3. Voiceover [SKIP TO PERIPH7A] 4. None |  |
| Q62 | PERIPH7A | If there is a voiceover, is it a real human voice or computer generated? | 1. Human voice 2. Computer-generated voice 3. Unclear |  |
| Q63 | PERIPH7B | If there is music, what type of music? | 1. Mainstream or popular song/music 2. TikTok generated musical sounds 3. Content creator original music 4. Unclear |  |
| Q64 | PERIPH8 | What type of video is it? | See CHARAC3 |  |
| Q65 | PERIPH9 | Is there a caption on the video besides the hashtags? | See CHARAC4 |  |
| Q66 | PERIPH10 | Does the video include people talking to others? | 1. No (0) 2. Yes (1) 3. Unclear (2) |  |
| Q67 | PERIPH11 | If any, what characteristics of fun are demonstrated? (Check all that apply; aside from instructional use) | 1. Playing 2. Laughing 3. Cheering 4. Decorating 5. Someone is saying there are having fun/good time, etc. 6. Other (free text) 7. None |  |
| Q68 | PERIPH12 | Does the video have high engagement? | See Section 3 data and calculate using the following formula:  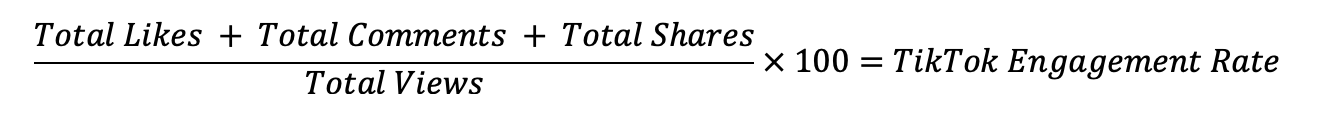 | Formula derived from: <https://keyhole.co/blog/tiktok-engagement-rate/#What-is-the-TikTok-engagement-rate-and-how-to-calculate-it> |
